# An Enhanced Bioactive Glass Composition with Improved Thermal Stability and Sinterability

**DOI:** 10.3390/ma17246175

**Published:** 2024-12-17

**Authors:** Andrea Martelli, Devis Bellucci, Valeria Cannillo

**Affiliations:** Department of Engineering “Enzo Ferrari”, University of Modena and Reggio Emilia, Via P. Vivarelli 10, 41125 Modena, Italy; andrea.martelli@unimore.it (A.M.); valeria.cannillo@unimore.it (V.C.)

**Keywords:** novel bioactive glasses, sintering optimization, heating microscopy, differential thermal analysis, crystallization, strontium, magnesium, potassium

## Abstract

The development of new bioactive glasses (BGs) with enhanced bioactivity and improved resistance to crystallization is crucial for overcoming the main challenges faced by commercial BGs. Most shaping processes require thermal treatments, which can induce partial crystallization, negatively impacting the biological and mechanical properties of the final product. In this study, we present a novel bioactive glass composition, S53P4_MSK, produced by a melt–quench route. This novel composition includes magnesium and strontium, known for their therapeutic effects, and potassium, recognized for improving the thermal properties of bioactive glasses. The thermal properties were investigated through differential thermal analysis, heating microscopy and sintering tests from 600 °C to 900 °C. These characterizations, combined with X-ray diffraction analysis, demonstrated the high sinterability without crystallization of S53P4_MSK, effectively mitigating related issues. The mechanical properties—elastic modulus, hardness and fracture toughness—were evaluated on the sintered sample by micro-indentation, showing high elastic modulus and hardness. The bioactivity of the novel BG was assessed following Kokubo’s protocol and confirmed by scanning electron microscopy, X-ray energy dispersive spectroscopy, and Raman spectroscopy. The novel bioactive glass composition has shown high sinterability without crystallization at 700 °C, along with good mechanical properties and bioactivity.

## 1. Introduction

The bioactive glass (BG) family emerged after the discovery of the first BG by Professor Larry Hench at the University of Florida in the 1970s [1]. Since then, many different compositions have been developed, and today various BGs are commercially available, particularly for dentistry and orthopaedics [2]. Among them, the most well-known compositions are 45S5 Bioglass^®^—the first to be developed, and used in more than 1.5 million patients—and S53P4, the first BG to exhibit antibacterial properties [1,3,4,5]. However, both glasses suffer from a narrow processing window, defined as the difference between the crystallization onset temperature (T_c_onset_) and the glass transition temperature (T_g_) [6,7]. This limitation poses a significant challenge due to the risk of crystallization during the thermal treatments which are required to obtain the sintered parts, such as scaffolds, or coatings from BG powders [8,9]. Indeed, partial crystallization during the sintering process can reduce the mechanical properties of the sintered part by inhibiting the sintering process itself; it can also negatively impact bioactivity and biological performance, owing to the different degradation rates of the amorphous and crystalline phases and the reduced ionic release once implanted in the body [6]. Crystallization can be partially limited with treatments involving high heating rates; however, it cannot be completely prevented in undoped glass [10]. Consequently, many studies have focused on addressing this issue by developing new compositions with different doping agents [11,12,13,14,15].

Among the various elements used as dopants, Mg and Sr have emerged as particularly interesting prospects, due to their biological properties and their influence on the physical properties of BGs [16,17,18,19,20,21,22], while K is especially promising for its effects on dissolution behaviour and physical properties [23,24,25]. Mg doping is known to decrease the T_G_ of BGs, as consequence of the lower strength of the Si-O-Mg bond compared to the Si-O-Si bond [26,27]. Indeed, Mg can act both as network-former and as an intermediate oxide [28,29]. Moreover, incorporating Mg into the BG composition can alter its mechanical properties [16,17,18], because Mg^2+^ has a higher ionic field strength compared to Ca^2+^ and Na^+^ [18]. Despite the fact that Mg-doping can lead to reduced hydroxyapatite formation [30], Mg-doped BGs showed excellent biocompatibility and osteogenic effects [19,20]. Similarly, excellent biological behaviour has also been found in Sr-doped BGs [22], although the effect of Sr on hydroxyapatite (HA) formation remains controversial [21,31]—HA precipitation is typically taken as an indicator of bioactivity for bioactive glasses. Additionally, the impact of Sr doping on the physical properties of BGs is also a subject of debate [32,33,34], as is the case for K-doped BGs [7,35]. In fact, when K_2_O is added to BG, it acts as a network modifier, and a high content of this oxide can significantly weaken the glass structure [36,37]. Conversely, partially substituting Na with K results in a significantly improved sintering window and a reduced tendency to crystallize, compared to undoped glass [7,23]. Furthermore, K doping can influence dissolution behaviour, affecting both apatite formation and the biological response [24,25]. 

Different studies have suggested promising results from the combined effects of Mg and Sr. In particular, research on BGs produced by the sol–gel technique for drug delivery has highlighted the influence of Sr content on particle size and demonstrated that the combined ion doping with Mg and Sr can affect drug loading and release, haemolytic activity, and cell proliferation in vitro [38,39]. Additionally, improved thermal stability—with a sintering temperature below the T_c_onset_—and good biological response, both in vitro and in vivo, were observed in a Mg-Sr doped composition derived from the “gold standard” 45S5 [40,41]. Similarly, the combined effects of Mg, Sr, and K were investigated in another BG composition, named BGMS10, which demonstrated excellent thermal stability, bioactivity, and biological performance both in vitro and in vivo [13,41]. 

These promising results encourage further investigation into new BG compositions containing Mg, Sr, and K. Here, we present and discuss a novel BG, containing these ions and with the same amount of P_2_O_5_ as the commercial S53P4, but with a lower sintering temperature and a higher crystallization temperature compared to its “parent” glass. This allows the BG composition to remain amorphous even after the sintering process. The novel glass, named S53P4_MSK, contains Mg, Sr, and K, along with a reduced Na content compared to typical silicate glasses such as 45S5 and S53P4, and appears particularly promising in terms of both sinterability and in vitro bioactivity.

## 2. Materials and Methods

**Figure 1:**
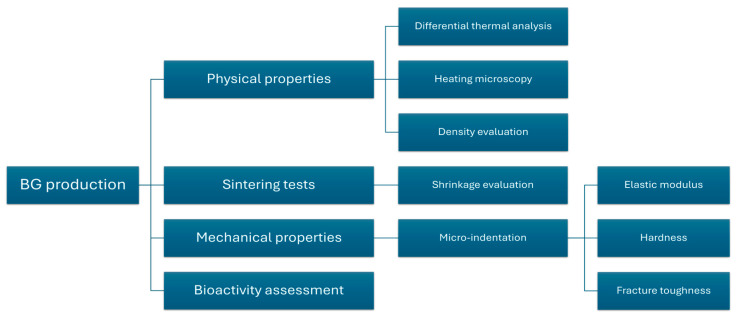
Flowchart of the experimental characterizations.

The analyses described in the following paragraphs evaluate the physical and mechanical properties of S53P4_MSK, as well as its sintering and bioactivity-based behaviours. Figure 1 presents a flowchart summarizing the experimental characterizations conducted.

### 2.1. Bioactive Glass Production

S53P4_MSK (composition, in mol%: 5.4 Na_2_O; 21.8 CaO; 2.3 K_2_O; 10.0 MgO; 10.0 SrO; 1.7 P_2_O_5_; 48.8 SiO_2_), was produced using a classical melt–quenching route, as described elsewhere [40]. High-quality raw materials in powder form (Carlo Erba Reagenti, Rodano-Milano, Italy) were carefully weighed and mixed for 2 h using a laboratory rotary mixer (MMS, Nonantola, Modena, Italy). The resulting mixture was melted in a platinum crucible following this thermal process: heating from room temperature to 1100 °C at a rate of 10 °C/min, decarbonation at 1100 °C for 1.5 h, further heating from 1100 °C to 1450 °C at 10 °C/min, and holding at 1450 °C for 1 h to achieve a homogeneous melt. The molten glass was rapidly quenched in water to form a frit, which was subsequently dried at 110 °C for 12 h. The obtained glass frit was ground in a ceramic jar and sieved under 63 μm for subsequent thermal analysis and sintering evaluation. 

### 2.2. Physical Properties

Thermal properties were evaluated on the BG powders using differential thermal analysis (DTA, Netzsch Differential Thermal Analyzer STA 429 CD, Netzsch-Gerätebau GmbH, Selb, Germany), heating the sample from room temperature to 1200 °C at a rate of 20 °C/min. Additionally, heating microscopy (HM, Misura 3.32; Expert System Solutions, Modena, Italy) was conducted from room temperature to 1600 °C, with a heating rate of 10 °C/min. The processing window was calculated as the difference between the onset crystallization (T_c_onset_) and the glass transition (T_g_) [6,7], while the sintering behaviour of S53P4_MSK was estimated by the sinterability parameter (S_c_) [42]:

(1) $$S_{c}=T_{c\_onset}-T_{s}$$

where *T_s_* is the sintering temperature.

The density of the BG powder was measured using a gas pycnometer (AccuPyc II 1340, Micromeritics Instrument Corp., Norcross, GA, USA). Helium with a total purity >99.999% mol was used as the dispersion medium.

### 2.3. Sintering Tests

Once the characteristic temperatures of S53P4_MSK were established, sintering evaluations were conducted on the glass powder pressed into disk form. The green bodies were obtained by pressing the powder in a mould using a hydraulic press (Mignon S, Nannetti, Faenza, Italy). These green bodies were then thermally treated for three hours at 600 °C, 700 °C, 800 °C, and 900 °C in a muffle furnace (AWF 13/12, Lenton—Laboratory Scientific Equipment, Randburg, South Africa) with a heating rate of 10 °C/min. The sintering behaviour of S53P4_MSK was investigated through volume shrinkage (Δ_%_) after thermal treatment. The following equation,

(2) $${∆}_{\%}=\frac{d_{0}-d_{s}}{d_{0}}\times 100$$

was used to calculate ∆_%_, where *d_0_* and *d_s_* are the nominal diameter of the mould and the measured diameter of the glass disks after sintering, respectively. All diameters were measured using a digital calliper (LTF 327.09, LTF S.p.A., Antegnate, Italy). Additionally, image analysis, using ImageJ software (v1.54g, NIH, Bethesda, MA, USA), was performed on the cross-sections of the disks to quantify residual porosity. The images were acquired using scanning electron microscopy (SEM) (Quanta 2000, FEI Co., Eindhoven, The Netherlands) (micrographs not presented, given brevity). Furthermore, the disks were ground and subjected to X-ray diffraction (XRD) analysis (X’Pert Pro, Panalytical, Almelo, The Netherlands) to detect any crystallization resulting from the thermal treatment. XRD scanning was conducted with Cu-Kα radiation over the range of 2θ = 10°–70° with increments of 0.017°.

### 2.4. Mechanical Properties

The elastic modulus and hardness (*HV*) of the sintered sample were assessed using the micro-indentation technique on a cross-section of the glass disks sintered at 700 °C. This temperature was chosen because it represents the temperature at which full sintering occurs with no crystallization. The evaluations were conducted utilizing the Open Platform equipment (CSM Instruments, Peseux, Switzerland), employing a Vickers indenter tip. The indentation process applied a 0.5 N load with a loading/unloading rate of 1 N/min, and the maximum load was sustained for 15 s. In sum, 15 measurements were taken, and an automatic recording of the load–penetration depth curve was made for each indentation. The elastic modulus was determined using the Oliver and Pharr method, which relies on the indentation load–unloading curves [43]. Additionally, fracture toughness was evaluated using a load of 1 N in order to make cracks propagate from the indent tips. Fracture toughness (*K_Ic_*) was then calculated based on the crack lengths according to widely used equations reported below (*EC*: Evans and Charles; *LF*: Lawn and Fuller; *EW*: Evans and Wilshaw; *L*: Lankford) [44,45]:

(3) $$K_{Ic,\;EC}=0.0824\times \frac{P}{c^{3/2}}\;\;$$

(4) $$K_{Ic,\;LF}=0.0515\times \frac{P}{c^{3/2}}\;\;$$

(5) $$K_{Ic,\;EW}=0.079\times \frac{P}{a^{3/2}}\times log\left(\frac{4.5\;a}{c}\right)$$

(6) $$K_{Ic,\;L}=0.0363\times {\left(\frac{E}{HV}\right)}^{2/5}\times \left(\frac{P}{a^{1.5}}\right)\times {\left(\frac{a}{c}\right)}^{1.56}$$

where *P* is the applied load, *c* is the average crack length measured from the indentation centre (in µm), *a* is the average indentation half-diagonal (in µm), *E* is the elastic modulus (in GPa), and *HV* is the Vickers hardness (in GPa).

### 2.5. Bioactivity Assessment

In vitro bioactivity assessments were conducted following the Kokubo protocol [46] on untreated BG granules ranging in size from 250 to 500 μm in simulated body fluid (SBF) at a concentration of 4% *w*/*v*. This specific grain size was chosen to control both the dissolution rate and ion release, in order to optimize the potential in vitro biological response [47]. The bioactivity tests were conducted at 37 °C, with time points set at 1, 3, 7, and 14 days. The pH measurements of the SBF were taken every 48 h, with fluid renewal occurring at the same interval. Once extracted, the granules were gently rinsed with distilled water to stop further reaction and dried at room temperature. Hydroxyapatite formation was evaluated by Raman spectroscopy (LabRAM Odissey, Horiba Jobin-Yyon, Villeneuve D’Aseq, France), using a device equipped with a 632.8 nm-wavelength laser. The surface morphology of the granules before and after immersion in SBF was examined using SEM with a field emission gun (FEG)—SEM (Nova NanoSEM 450, FEI Co., Eindhoven, The Netherlands). Further characterization of apatite precipitation was performed using X-ray energy dispersive spectroscopy (EDS) (Inca, Oxford Instruments, Abingdon, UK).

## 3. Results and Discussion

### 3.1. Physical Properties

**Figure 2:**
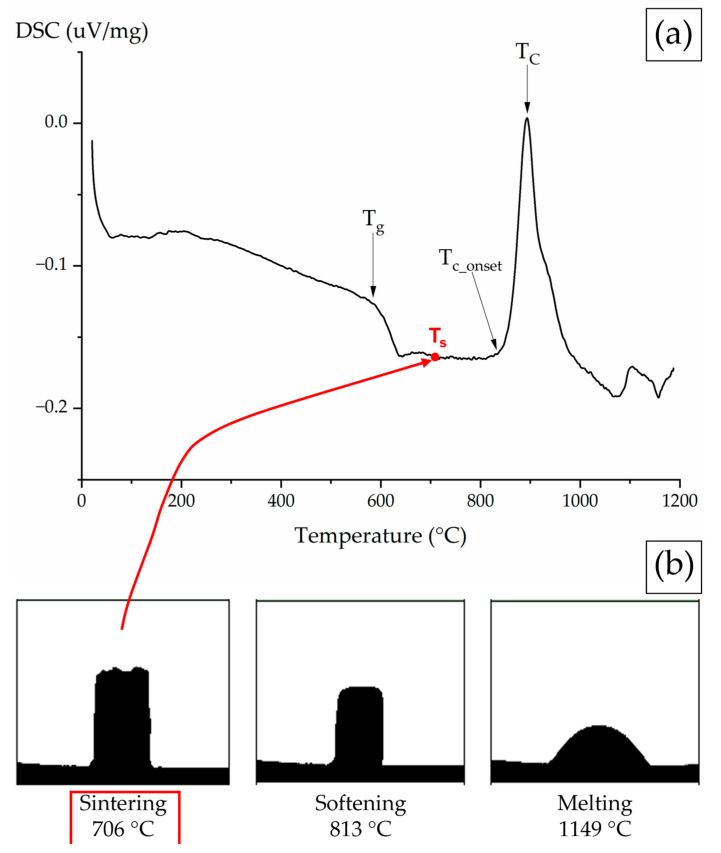
(**a**) DTA graph and (**b**) HM of S53P4_MSK glass. The characteristic temperatures T_g_, T_s_, T_c_onset_, and T_c_ are shown on the DTA curves.

The data obtained from DTA and HM are shown in Figure 2. The HM output consists of a series of images acquired during the sample’s heat treatment. Using geometric calculations, HM can determine temperatures critical for sintering, softening, and melting [48]. These values, along with the results from the density analysis for S53P4_MSK, are summarized in Table 1 alongside those of commercially available 45S5 and S53P4 [48], provided for comparison.

S53P4_MSK begins crystallization above 835 °C, reaching its peak at 894 °C. This high crystallization temperature shows promise for tissue engineering applications that require thermal treatments like scaffolds or coatings, due to the wider processing window it offers. In contrast, the two most commonly used BGs, as a comparison, exhibit lower crystallization temperatures and narrower processing windows. Specifically, 45S5 has a crystallization temperature (T_c_) of 676 °C [51], and S53P4 has a T_c_ of 730 °C [49]. Furthermore, as indicated in Table 1, both of the commercial BGs exhibit negative S_c_ values, highlighting their tendency to crystallize during the sintering process. The enhanced thermal stability of S53P4_MSK could be attributed to (i) the lower content of Na_2_O, which is known to induce crystallization [54]; (ii) the potential inhibitory effect of MgO on devitrification [55]; and (iii) the increased entropy of the system, favouring the amorphous condition [56].

### 3.2. Sintering Tests

To further evaluate the sintering behaviour of S53P4_MSK, both volumetric shrinkage and the density of disk cross-sections treated at different temperatures were analyzed. The results are summarized in Table 2. The sintering process identified from the data reveals that densification begins between 600 °C and 700 °C, reaching its maximum at this point, and continues up to 800 °C. Afterward, densification decreases in terms of both shrinkage and density, possibly due to crystal nucleation and growth. 

**Figure 3:**
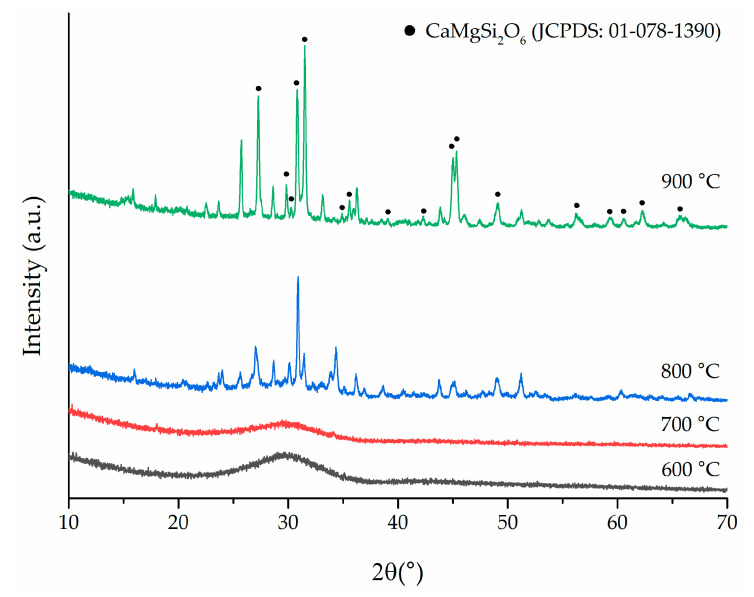
XRD diffractograms of S53P4_MSK after thermal treatment for 3 h at different temperatures ranging from 600 °C to 900 °C. The primary crystalline phase identified is diopside (CaMgSi_2_O_6_, JCPDS no. 01-078-1390).

XRD diffractograms (Figure 3) acquired from samples treated at different temperatures confirmed the crystallization of S53P4_MSK starting at 800 °C, while showing the typical pattern of an amorphous glass at temperatures equal to or lower than 700 °C. The impact of ion doping with Mg, Sr, and K, coupled with the concurrent reduction in Na content, is readily apparent, as no signs of crystallization are detectable at the sintering temperature. This outcome can be attributed to the previously reported effects of composition on thermal stability [54,55,56]. Typically, heat treatment of S53P4 leads to the formation of sodium-calcium silicate, although the stoichiometry of the formed crystalline phase remains a subject of debate [57,58]. Despite the reduced sodium content, the analogous formation of sodium-calcium silicate (Na_2_CaSiO_4_) was detectable in S53P4_MSK at 800 °C. Moreover, the crystallization of the novel S53P4_MSK composition resulted in the formation of other phases at T_c_, whose identification was challenging due to peak overlap. Among these, diopside (CaMgSi_2_O_6_, JCPDS no. 01-078-1390) could be identified, as reported elsewhere for crystallization of Mg-doped BGs [59]. 

It is important to note that the DTA and XRD results may appear somewhat contradictory regarding the temperature at which the crystallization process begins. In fact, while the DTA reported a T_c_onset_ of 835 °C, XRD already detected crystallization at 800 °C. This discrepancy is due to the different thermal treatments applied: in the case of DTA, where T_c_onset_ and T_c_ are measured, the samples were simply heated to melting at 20 °C/min, while the samples analyzed with XRD were sintered with a thermal treatment lasting three hours at the final temperature. This prolonged treatment triggered crystallization, even though the temperature was slightly lower than T_c_onset_.

### 3.3. Mechanical Properties

The mechanical properties of S53P4_MSK were evaluated on samples thermally treated at 700 °C, the temperature at which full sintering with no crystallization occurs. The results derived from the indentation are reported in Table 3. The findings revealed that the elastic modulus and hardness of the S53P4_MSK are slightly higher than those reported in analogous studies [6,60,61]. This can be attributed to the addition of Mg, Sr, and K ions and the lower content of Na ions. In fact, these ions can occupy the interstitial sites within the ceramic-glass networks, improving the mechanical properties of the BGs [60]. Moreover, the fracture toughness results are consistent with the range reported for BGs in the literature [62].

### 3.4. Bioactivity Assessment

**Figure 4:**
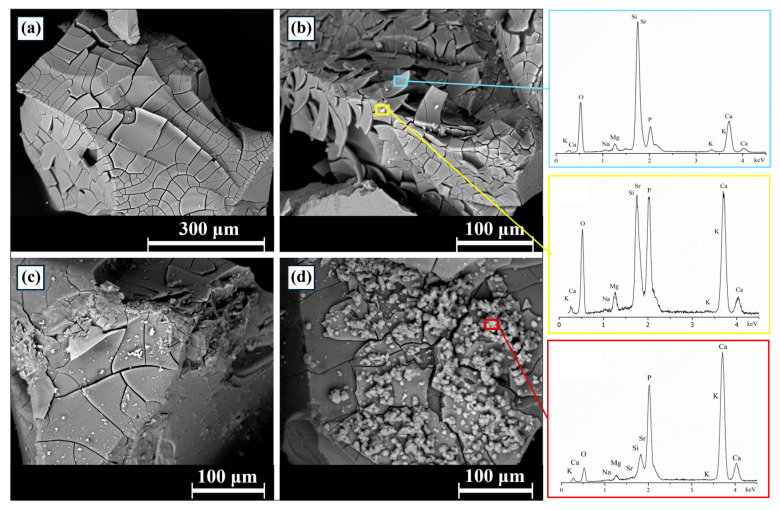
SEM micrographs of S53P4_MSK granules after (**a**) 1, (**b**) 3, (**c**) 7, and (**d**) 14 days of soaking in SBF. On the right are shown the EDS analyses of the highlighted areas in the micrographs.

**Figure 5:**
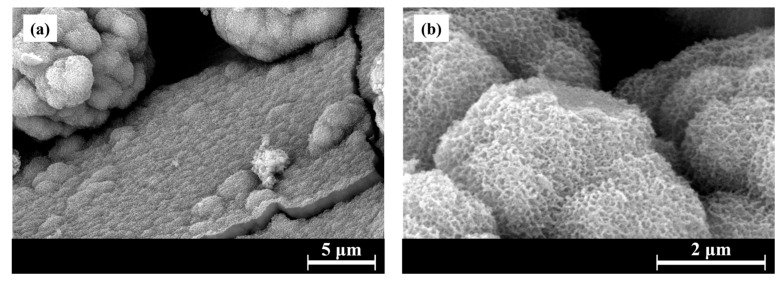
FEG-SEM micrographs of S53P4_MSK granules after 14 days of soaking in SBF.

The bioactivity of S53P4_MSK was assessed in vitro using simulated body fluid solution (SBF) over a period of up to 14 days. SEM analysis showed that the surface of S53P4_MSK granules begins to convert into HA within one day (Figure 4a), a process marked by the formation of a silica gel [63]. The silica gel appears cracked due to the drying of the samples [64]. Globular precipitates were observed after three days (Figure 4b). This morphology is characteristic of in vitro-formed apatite [65,66]. High-magnification acquisition shows the typical cauliflower-like structure of apatite formation after 14 days of soaking in SBF (Figure 5). The high concentrations of calcium and phosphorus found in the EDS analysis of these structures support the hypothesis that apatite formation is occurring on the surface of the granules [67]. 

**Figure 6:**
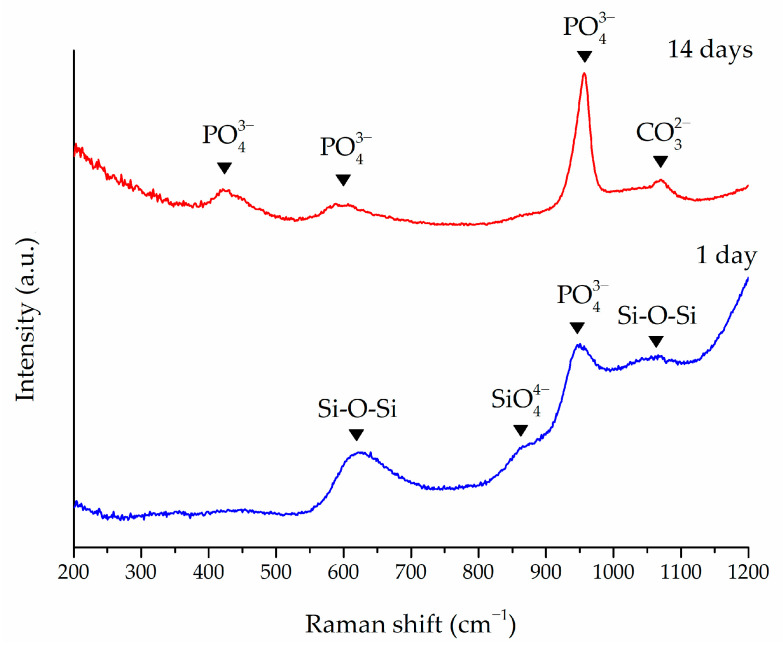
S53P4_MSK Raman spectra of granules soaked in SBF for 1 or 14 days.

The in vitro bioactivity of the novel glass has been further confirmed using Raman spectroscopy. Figure 6 presents the spectra acquired on BG granules after 1 and 14 days of soaking in SBF. The spectrum after 1 day revealed the presence of peaks associated with silicate groups [68] and phosphate groups present in the original structure of the BG [69]. In particular, the broad peak around 635 cm^−1^ is attributed to the Si-O-Si bond, while the partially obscured peak at 860 cm^−1^, appearing as a shoulder due to the main peak around 950 cm^−1^, represents the monomer SiO_4_^4−^. The main peak around 950 cm^−1^ is commonly associated with the symmetrical stretching of the PO_4_^3−^ group [64]. Finally, the peak around 1075 cm^−1^ can be attributed to the asymmetrical stretching of the Si-O-Si group [70]. These features are commonly associated with a BG that has not yet reacted [64]. 

Conversely, the spectra acquired after 14 days of soaking show peaks commonly associated with in vitro-formed HA. The peaks related to silicate groups become less intense, yielding to the increased intensity of the phosphate- and carbonate-group peaks. Specifically, the peaks associated with the phosphate group that indicate PO_4_^3−^ bending are observed around 430 and 590 cm^−1^ [71,72] and are accompanied by a shift of the main peak from 950 to 960 cm^−1^ [64]. Additionally, the peak at 1070 cm^−1^ signifies the stretching of a carbonate group [73], suggesting the formation of hydroxyl-carbonate apatite (HCA) [13]. HCA is a compound chemically and structurally similar to HA that represents a preliminary step for bio-integration [64,74,75].

Alongside its bioactivity, the novel composition exhibits optimal reactivity in vitro, as indicated by pH measurements ranging from 7.75 to 8.1 throughout the duration of the experiments. This finding is highly promising, as mildly alkaline environments are widely recognized as beneficial for the promotion of tissue regeneration by stimulating osteogenic activity and inducing the antibacterial effects of BGs [76,77,78]. Moreover, such minor pH fluctuations are expected to minimize potential cytotoxic effects. Significant pH changes can indeed damage cells, underscoring the necessity to precondition certain BG compositions before cell exposure [79].

## 4. Conclusions

One of the main challenges for BGs lies in their low thermal stability, which often results in crystallization during the thermal treatments required for coating or sintering processes. While using a high heating rate can partially mitigate this issue, it cannot completely prevent crystallization [10]. One alternative approach involves the use of doping agents to expand the sintering window. In this context, S53P4_MSK is a new BG composition doped with Mg, Sr, and K, and retaining the same P_2_O_5_ content as the commercial S53P4.

The main advantages of S53P4_MSK include the following:

- High sinterability at 700 °C with no crystallization.
- High bioactivity, with HA formation observed after 3 days in SBF.
- Good mechanical properties.
- The presence of biologically significant ions.

The combination of these physical properties with the material’s potential biological benefits makes this novel BG particularly promising for applications requiring thermal treatments. Future studies will focus on the biological performance of this new composition, including investigations into its potential angiogenic, osteogenic, and antibacterial properties. Additionally, the production of sintered scaffolds will be explored to further evaluate the biological performance of 3D structures made with S53P4_MSK.

## Figures and Tables

**Table 1:** Characteristic temperatures and density of S53P4_MSK glass compared to those of S53P4 and 45S5.

	T_g_ (°C)	T_s_ (°C)	T_c_onset_ (°C)	T_c_ (°C)	T_m_ (°C)	S_c_ (°C)	Processing Window (°C)	Density (g/cm^3^)
S53P4_MSK	600	706	835	894	1149	129	235	2.957 ± 0.001
S53P4 [49,50]	520	700	650	730	1100	<0	130	2.66
45S5 [51,52,53]	549	1050	610	676	1165	<0	61	2.70

**Table 2:** Sintering behaviours of S53P4_MSK at different temperatures.

Temperature (°C)	600	700	800	900
Shrinkage (%)	0.10 ± 0.06	14.78 ± 0.12	14.55 ± 0.12	6.66 ± 0.09
Density (%)	NA	99.68 ± 0.05	99.48 ± 0.08	95.58 ± 0.17

**Table 3:** Mechanical properties of S53P4_MSK. *EC*: Evans and Charles; *LF*: Lawn and Fuller; *EW*: Evans and Wilshaw; *L*: Lankford.

Elastic Modulus (GPa)	HV (GPa)	K_IC_ (MPa m^1/2^)			
		*EC*	*LF*	*EW*	*L*
91.38 ± 2.81	6.91 ± 0.35	0.70	0.44	0.65	0.79

## Data Availability

The original contributions presented in this study are included in the article. Further inquiries can be directed to the corresponding author.

## Abbreviations

BG	Bioactive glass
DTA	Differential thermal analysis
EC	Evans and Charles
EDS	X-ray energy dispersive spectroscopy
EW	Evans and Wilshaw
FEG	Field emission gun
HA	Hydroxyapatite
HM	Heating microscopy
HV	Hardness
K_I_c	Fracture toughness
L	Lankford
LW	Lawn and Fuller
SBF	Simulated body fluid
Sc	Sinterability parameter
SEM	Scanning electron microscopy
T_c_	Crystallization temperature
T_c_onset_	Crystallization onset temperature
T_g_	Glass transition temperature
XRD	X-ray diffraction
Δ_%_	Volume shrinkage
